# Developing a hybrid FRP-concrete composite beam

**DOI:** 10.1038/s41598-022-20666-x

**Published:** 2022-09-28

**Authors:** Mantas Garnevičius, Viktor Gribniak

**Affiliations:** 1 Department of Steel and Composite Structures, Vilnius Gediminas Technical University (VILNIUS TECH), Sauletiekio av. 11, 10223 Vilnius, Lithuania; 2 Laboratory of Innovative Building Structures, VILNIUS TECH, Sauletiekio av. 11, 10223 Vilnius, Lithuania

**Keywords:** Civil engineering, Composites

## Abstract

Current materials engineering trends put forward the development of efficient structural solutions. The steel replacement with fiber-reinforced polymers (FRP) exemplifies the key to the corrosion problem. However, the relatively low deformation modulus of typical FRP materials raises the deformations of the structural components. Together with the self-weight reduction increasing the kinematic displacements, the latter issue makes developing hybrid structures comprising compression-resistant concrete and high-performance in tension FRP profiles important. Although such hybrid systems are applicable for bridge engineering, the uncertainty of the inter-component bonding properties complicates developing these innovative structures, including the design models. The typical solution focuses on the local bond improvement, e.g., employing FRP profile perforation and mechanical anchorage systems. However, this study introduces an alternative solution, using the stress-ribbon bridge structural system for creating the hybrid beam prototype, which combines the synthetic fiber-reinforced concrete slab and pultruded FRP profile fixed on the supports. This work exemplifies the structural development concept when the finite element (FE) modeling outcome defines the target reference of the design procedure. Thus, on the one hand, this innovative structure simplifies the corresponding numerical (FE) model, which assumes the perfect bond between the components of the hybrid beam system. On the other hand, the solution to the support problem (resulting from a low resistance of pultruded FRP profiles to transverse loads) improves the structural performance of the bridge prototype, doubling the structure’s flexural stiffness and load-bearing capacity regarding the weak concrete supports’ system. The bending tests proved the adequacy of this solution in describing the design reference for further development of the proposed structural concept.

## Introduction

The materials engineering trends put forward the development of efficient structural solutions^1,2^. As a result, there is a tendency to develop new structural materials to change traditionally used concrete and steel^3^. Fiber-reinforced polymers (FRP) define the promising alternative to steel, and carbon, glass, and aramid fiber-based composites are the most common FRPs on the market^4,5^. It is known that manufacturing technology affects the mechanical performance of FRP composites. Thus, this study focuses on the pultruded objects because of the ability of the pultrusion technologies to produce a large volume at low operating costs and high fabrication rate, fiber content, and geometry tolerances^6,7^.

The pultrusion direction and reinforcement filament distribution coincide, ensuring the mechanical performance of the structural FRP parts^6–9^. However, such components often face transverse loads regarding the pultrusion pathway; moreover, the pultruded details must resist bolt removal-induced local stresses^4,5^. Therefore, the smooth unidirectional roving and mats protect the longitudinal filaments, complicating the internal reinforcement structure of the FRP material^6^. At the same time, these additional protection means can be insufficient for developing FRP structures^10–12^. In addition, the relatively low deformation modulus of typical FRP materials raises the deformations of the structural components. Together with the self-weight reduction increasing the kinematic displacements^13^, the latter issue makes developing hybrid structures comprising compression-resistant concrete and high-performance in tension FRP profiles important.

Although hybrid composite systems are applicable for bridge engineering^13–15^, the uncertainty of the inter-component bonding properties complicates developing these innovative structures. The typical solution focuses on local bond improvement, employing FRP profile perforation and mechanical anchorage systems, e.g., Mendes et al.^16^ and Zhang et al.^17^. However, the design of such structures lies beyond the standard regulation field. At the same time, the bond problem complicates structural analysis and numerical modeling^18,19^. Still, studies^9,20–23^ describe the typical analysis examples, neglecting the bond problem.

References^8,24–27^ define the cases when the bond parameters were among the research subjects. For instance, Chen et al.^26^ focused the research on the FRP laminate bonding properties. Four remaining works take into account the FRP-concrete bond performance of the hybrid structural systems, which describe the research object of this study. Dang and Phan^8^ and Cai et al.^25^ investigated the FRP bar bonding performance in concrete. Robinson and Melby^24^ studied the mechanical resistance of the concrete-filled GFRP tube, and Muc et al.^27^ simulated the composite deck slab. However, a rare publication considers the support joint’s resistance of the FRP profiles, e.g., Zhang et al.^28^.

In contrast, this study employs the stress-ribbon bridge solution to create the hybrid beam prototype, combining the polymeric fiber-reinforced concrete (PFRC) slab and pultruded FRP profile. However, the proposed structural system does not require massive supports typical for the stress-ribbon systems^15^ because of the combination of the concrete, resisting the compressive load induced by the glass-fiber-reinforced profile (GFRP) distributed in the tension zone of the flexural element. Furthermore, the reliable profile fixation to the supports ensures the composite behavior of the hybrid beam. In addition, it simplifies the corresponding finite element (FE) model, allowing the perfect bond assumption between the composite parts. Thus, this FE model describes the reference for developing the hybrid beam system. The bending tests substantiate the solution adequacy and exemplify the situation when the numerically predicted outcome determines the hybrid system efficiency and provides the designer with the structural reference.

## Results—the hybrid beam concept

### A preliminary structural scheme

**Figure 1:**
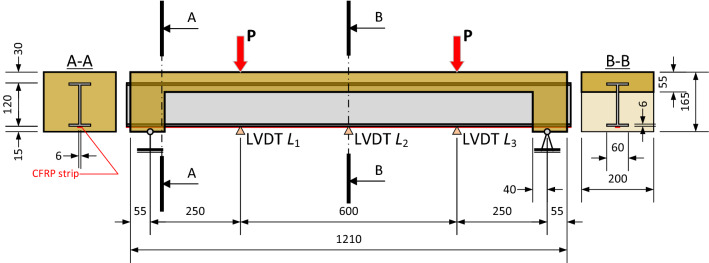
The hybrid beam schematic and anticipated cross-sections.

The designed beam comprises the polymeric fiber-reinforced concrete (PFRC) slab, resisting the compression force from the support joints and fixing the glass-fiber-reinforced profile (GFRP, 120×60/6/6 mm I-profile by Fiberline, Denmark). The preliminary simulations^29^ determined the support block’s geometry. In addition, a carbon-fiber-reinforced polymer (CFRP, 10 × 1.4 mm by S&P C-Laminate, UK) strip strengthens the most tensioned face of the GFRP profile. Figure 1 demonstrates the composite beam’s schematic and anticipated cross-sections.

The structural scheme (Fig. 1) employs the stress-ribbon bridge concept^15,30^ to develop the hybrid beam. In such a way, the concrete provides a reliable connection with the GFRP profile on the supports. In addition, FE modeling checks the proposed concept’s viability when the smeared reinforcement approach^7^ describes the mechanical performance of FRP components, the physically nonlinear material model^31^ defines the PFRC behavior, and the perfect bond model represents the contact problem.

### Numerical model

**Figure 2:**
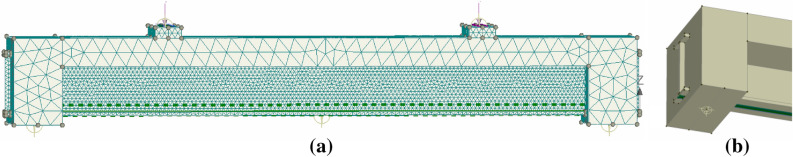
The finite element model of the hybrid beam: (**a**) FE discretization; (**b**) support view.

The commercial software Atena helps analyze deformation response and predict the load-bearing capacity of the hybrid beam; the material models verified in the previous works^1,7,31^ describe the mechanical behavior of PFRC and FRP components. The tetrahedral mesh generates the finite element (FE) model, shown in Fig. 2. The protective elastic plates on the load application points (Fig. 2a) have a 15 mm FE mesh. The monolithic concrete part of the beam (Fig. 2b) has a 30 mm mesh size; the CFRP strip and GFRP profile have a 7.5 mm finite element size. The model monitoring results are vertical displacements at the mid-span and load application points.

The Non-Linear Cementitious material model with 55 MPa compressive strength^31^ determines the deformation behavior and failure mechanism of PFRC. An elastic-plastic model (elasticity modulus = 170 GPa and tensile strength = 2.8 GPa) determines the material behavior of the 10 × 1.4 mm CFRP strip^1^. The 3D solid finite elements describe the polymer matrix of the GFRP profile, assuming the fracture mechanic principles for tensile failure and the plasticity approach to compressive failure.

Gribniak et al.^7^ adapted the smeared reinforcement model, initially developed for reinforced concrete elements with structural mesh reinforcement^32^, to represent the glass filaments and verified this numerically efficient solution, simulating the three-point-bending tests of FRP profile fabricated by the same manufacturer as this study. The verification^7^ demonstrated the FE model’s ability to predict the profile’s load-bearing capacity and deformation response. An elastic-brittle constitutive law defines tension failure of the fibers oriented in the pultrusion direction. A 63.4% smeared reinforcement ratio was assumed for this analysis, corresponding to the previous research^7^. The polymer matrix has an elastic modulus of 3.23 GPa, and tensile strength of 90 MPa. The E-glass fibers (smeared reinforcement) have a 73 GPa elastic modulus and a 3445 MPa tensile strength; the filaments do not resist compression stresses. The perfect connection was assumed between all the model components. The first loading stage considers the self-weight of the beam. Two point-loads were applied in succeeding increments resulting in a 0.125 kNm moment increase at each successive loading increment on the 600 mm pure bending zone (Fig. 1). Physical tests verify the model adequacy.

### Physical tests of the beam prototypes

The physical tests were carried out in two stages, and two nominally identical beams were produced for each series. The specimens were poured using the same mix proportions with a target 55 MPa compressive strength, as considered in the previous studies^1,31^. The following mix proportions for one cubic meter were used: 356 kg of cement CEM I 42.5 R; 201 l of water; 177 kg of limestone powder; 890 kg of 0/4 mm sand; and 801 kg of 4/16 mm crushed aggregates. The concrete also included 2.61% of the cement weight of the superplasticizer Mapei Dynamon XTend and 3.5 kg of the admixture SCP 1000 Optimizer. In addition, the mixture included 4.2 kg of macro-fibres Durus EasyFinish and 0.6 kg of micro-fibres CrackStop M Ultra by Adfil NV (Belgium).

The construction of the support joints made the difference between the beam series. The first series produced the hybrid beam prototypes with nominal geometry, shown in Fig. 1. The insufficient resistance of the supports in fixing the GFRP profile motivated the development of the second beam series. Two rectangular 550 × 550 × 40 mm slabs and eight 100 mm cubes were produced together with the hybrid beams of each series. The vibration table densified the concrete structure. Perforations were located in the web of the support zone and the top flange of the GFRP profile to improve the contact performance with PFRC in beams belonging to the first series. On the contrary, the second series profiles had no perforation.

All the beams were poured into the inverted position. Steel forms with plywood planks were utilized for the beams’ production. The foamed polystyrene and wood inserts were used to form the support blocks, fixing the GFRP profile. The concrete was poured into two layers. In the first layer, a 55 mm thickness concrete deck was formed and densified using the vibration table. After that, the concrete supports were formed using foamed polystyrene plugs. The support blocks of the first beam series ensure the 20 mm cover of the profile on the beam support. The 95 mm monolithic concrete support blocks (Fig. 1) were densified by poking them with a metal rod.

**Figure 3:**
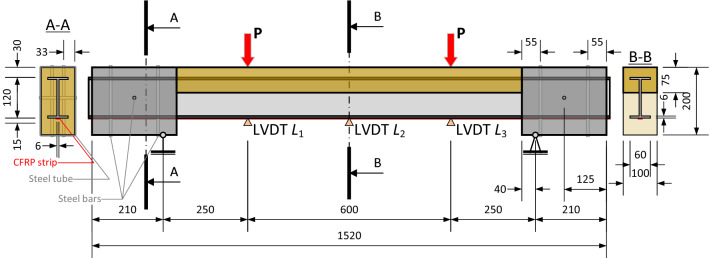
The updated hybrid beam schematic and modified cross-sections.

**Figure 4:**
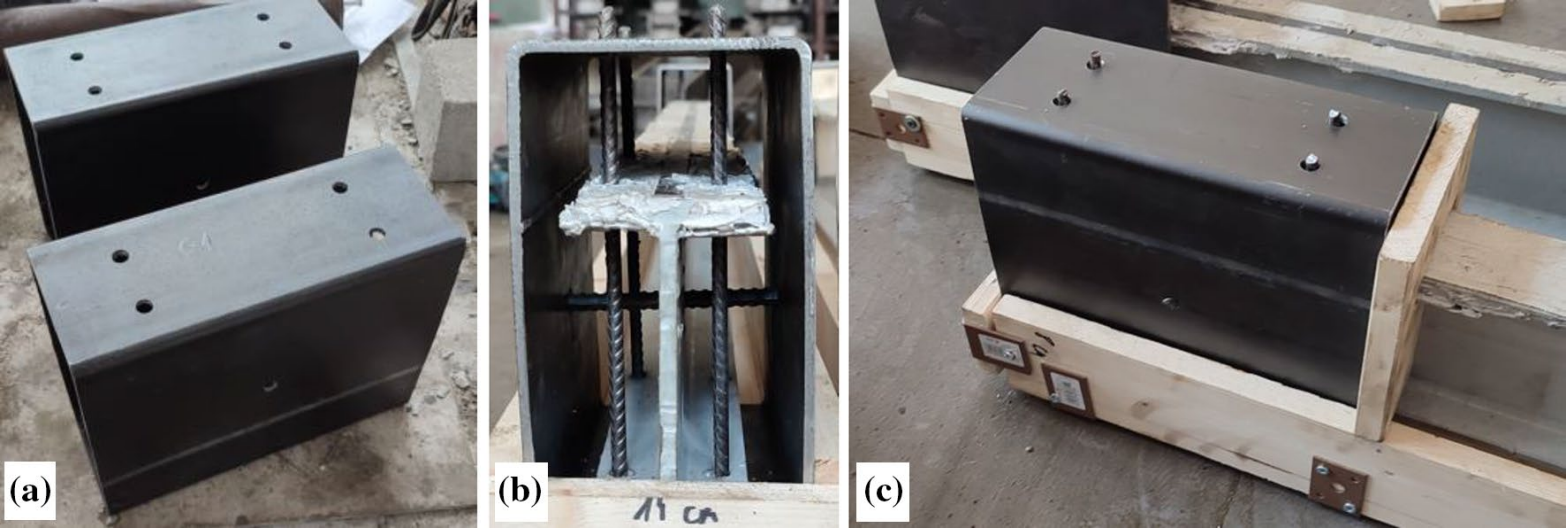
Preparing the modified support blocks: (**a**) rectangular steel tube with drilled holes; (**b**) inserted 6 mm and 8 mm bars; (**c**) the steel tube inside the form.

The beam specimens from the second group had improved anchorage blocks—a hollow-section steel 100 × 200 mm rectangular profile protected the GFRP profile at the supports. This modification increased the support block length from 95 to 250 mm, but the support distance remained the same. In addition, the width of the compressive concrete zone was decreased to simplify the beam production, preserving the flexural rigidity of the beam by increasing the compressive zone height. The remaining components of the hybrid system, i.e., GFRP profile, CFRP strip, and adhesive, remained the same. Figures 3 and 4 show the beam schematic and the anchorage block views.

The vertical 6 mm and horizontal 8 mm steel bars prevented movements of the GFRP profile inside the steel tube (Fig. 4). The 8 mm bar went horizontally through the middle of the GFRP profile (Fig. 3). In the same way as for the first series beams, two beam prototypes, two 550 × 550 × 40 mm slabs, and eight 100 mm cubes were produced using identical concrete proportions (described in this section, above). The beam samples were poured into two layers: the first layer, including the concrete slab, was densified using the vibrating table, and the second layer formed the support blocks. This concrete was carefully distributed and densified inside the protective steel tubes using steel rods.

**Figure 5:**
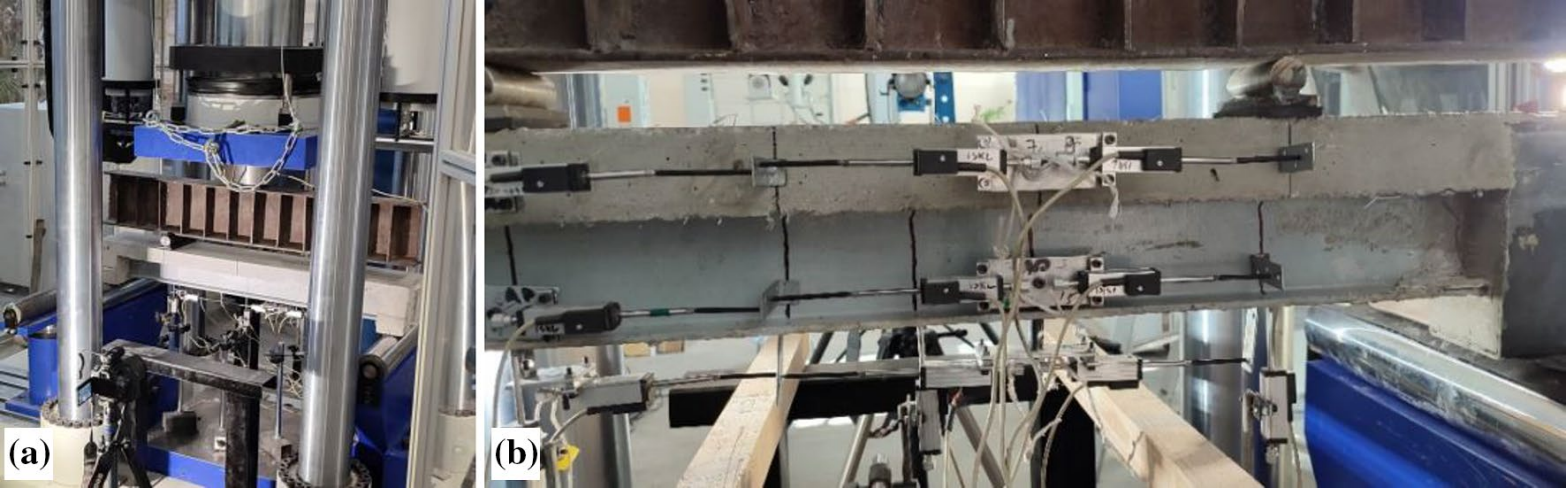
Examples of the bending test setup of the hybrid beam: (**a**) the surface exposed for digital image correlation; (**b**) distribution of linear variable displacement transducers, monitoring vertical and horizontal displacements.

The beams of both series were demolded after two days and stored in laboratory conditions (average temperature 20 °C and 40% relative humidity) for 30 days before the tests. The same loading scheme and distribution of the measurement devices were used for all tested beams. Figure 5 shows the characteristic views of the bending test setup. A digital image correlation system (Fig. 5a) was used to capture a sudden failure of the hybrid beam specimens. However, the manuscript does not include these results because of the gradual collapse of the beam samples. In addition, the uneven surface of the beams made the image correlation procedure inefficient for capturing deformation responses. Thus, this study employs the linear variable displacement transducers (LVDT) to capture the vertical displacements at the beam mid-span and below the load application points (Fig. 5b). In addition, nine LVDT monitored surface deformations in the pure bending zone. Then, they were applied to monitor longitudinal strains within the bending zone.

The bending tests were carried out using a 5 MN servohydraulic machine with the load applied in the displacement control manner with the 0.4 mm/min rate. A load cell was used to measure the applied load. An ALMEMO 2890-9 data logger recorded the reading of all LVDT devices and the load cell. The outputs were collected every second.

## Discussion

The compressive tests identified 65.6 MPa and 70.3 MPa strength of 100 mm concrete cubes, which correspond to the 49.9 MPa, and 53.4 MPa strength of the standard ∅150 × 300 mm cylinders^33^. These results reasonably agree with the assumed 55 MPa target strength of PFRC. In addition, the test results of the punching shear tests of the 550 × 550 × 40 mm slabs verified the PFRC material model, and these results are not included in this article.

### Moment–curvature response of the hybrid beams

The moment-curvature response describes the adequate measure of the global deformation behavior of the composite beams^1,7,33^. The analysis employs the vertical displacements’ monitoring results of the pure bending zone (Fig. 5b). The following formula defines the curvature over the pure bending zone, assuming the circular deformation shape of the beam centerline^1^:

1 $$\begin{array}{*{20}c} {\kappa = \frac{8 \cdot \delta }{{l_{b}^{2} + 4 \cdot \delta^{2} }},} & {\delta = L_{2} } \\ \end{array} - \left( {L_{1} + L_{3} } \right)/2,$$

where *l*_*b*_ is the length of the pure bending zone (= 600 mm); *L*_1_, *L*_2_, and *L*_3_ are the LVDT readings (Figs. 1 and 5b).

**Figure 6:**
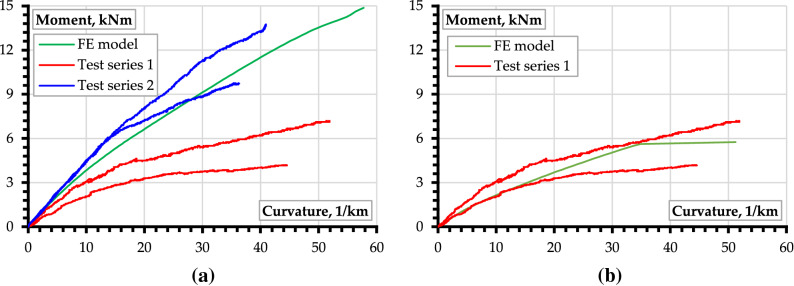
The experimental moment–curvature relationships of the hybrid beams compared with numerical predictions: (**a**) the reference model with the perfect bond between the composite components; (**b**) the model assumed a weak contact between the GFRP profile and concrete support blocks.

Figure 6 shows the corresponding moment–curvature diagrams from the physical test results and numerical simulations. In this study, the FE modeling outcomes define the reference for developing an efficient hybrid beam system. Let us consider the first series of results shown in Fig. 6a. The insufficient strength and stiffness of the hybrid beam (Fig. 1) are evident. Thus, the authors decided to reduce the bond strength (≈ 10 Pa) between the FRP components (GFRP profiles and CFRP strips) and concrete supports (Fig. 2b). Figure 6b shows the corresponding simulation results demonstrating perfect agreement between the FE model prediction and the test outcomes. This fact stimulated the modification of the support blocks (Fig. 3). The blue lines in Fig. 6a illustrate the deformation response of the second beam series. The test outcomes reveal noticeable agreement with the FE model, which was considered an efficient reference, assuming the perfect bond between all composite parts of the hybrid beam. Moreover, the second series of beams had no perforation of the GFRP profile.

Remarkably, Fig. 6 demonstrates the conceptual example of the proposed design philosophy when an experimentally verified numerical model describes the target reference. The detailed analysis requires additional tests (to ensure the reliability of the numerical estimations). However, the differences between the alternative solutions shown in Fig. 6a,b are apparent, illustrating the concept’s efficiency. Thus, the results of the second beam series (Fig. 6a) allow relating the improvement of the structural performance with the proposed modification of the support joints, anchoring the GFRP profile. In addition, this solution simplifies the numerical model—the perfect bond assumption solves the modeling problems reported in the literature^27^, making the FE approach acceptable for designing the hybrid systems considered in this study.

### Longitudinal strain distribution in the bending zone

**Figure 7:**
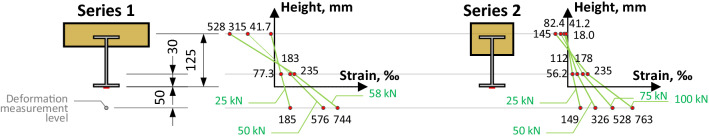
Longitudinal strain distribution in the hybrid beams.

Figure 5b shows the longitudinal strain gauges’ arrangement. It can be observed that the LVDT devices were distributed in three lines with a 50 mm offset of the bottom line regarding the bottom surface of the GFRP profile. Figure 7 shows the deformation profiles corresponding to the beam reaction monitored with the load-cell—the strain results from averaging three LVDT devices distributed in the row (Fig. 5b). The 58 kN load defines the failure of the beams belonging to the first series.

The strain distribution in Fig. 7 is close to linear and is characteristic for all loading stages and both series of beams. This outcome substantiates the slip absence between the hybrid beam components, proving the adequacy of the perfect bond assumption in the numerical model. At the same time, the inefficient behavior of the first series of beams (Fig. 6b) requires clarification, considering the beam failure mechanisms.

### Failure mechanisms of the hybrid beams

**Figure 8:**
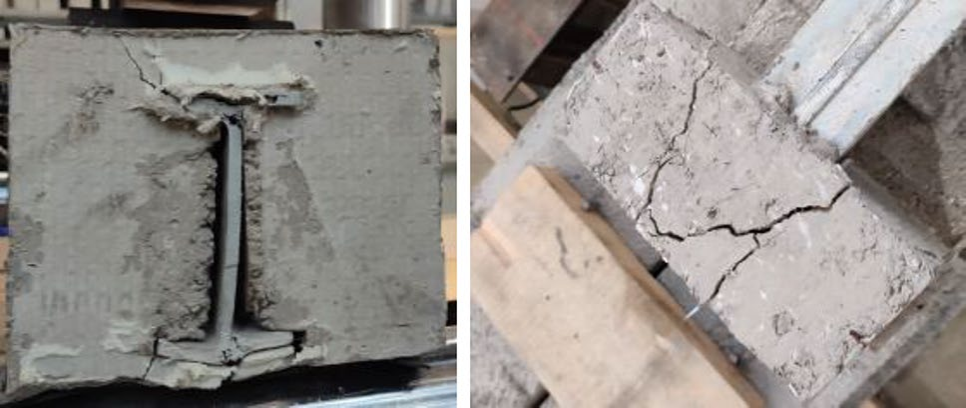
The typical failure of the support block of the first series beam.

The crushing of the GFRP profile anchorage blocks results from the first series of tests. Figure 8 shows a typical view of the beam support after the collapse. This outcome is a consequence of the insufficient resistance of the FRP materials to the transverse loads to the pultrusion direction, which corresponds to the literature results^6,28,30^. However, the FE model could not represent this failure mechanism because of the limited ability to simulate the transverse crushing of FRP materials (Fig. 6a), resulting from the heterogeneity of the material structure^7^.

**Figure 9:**
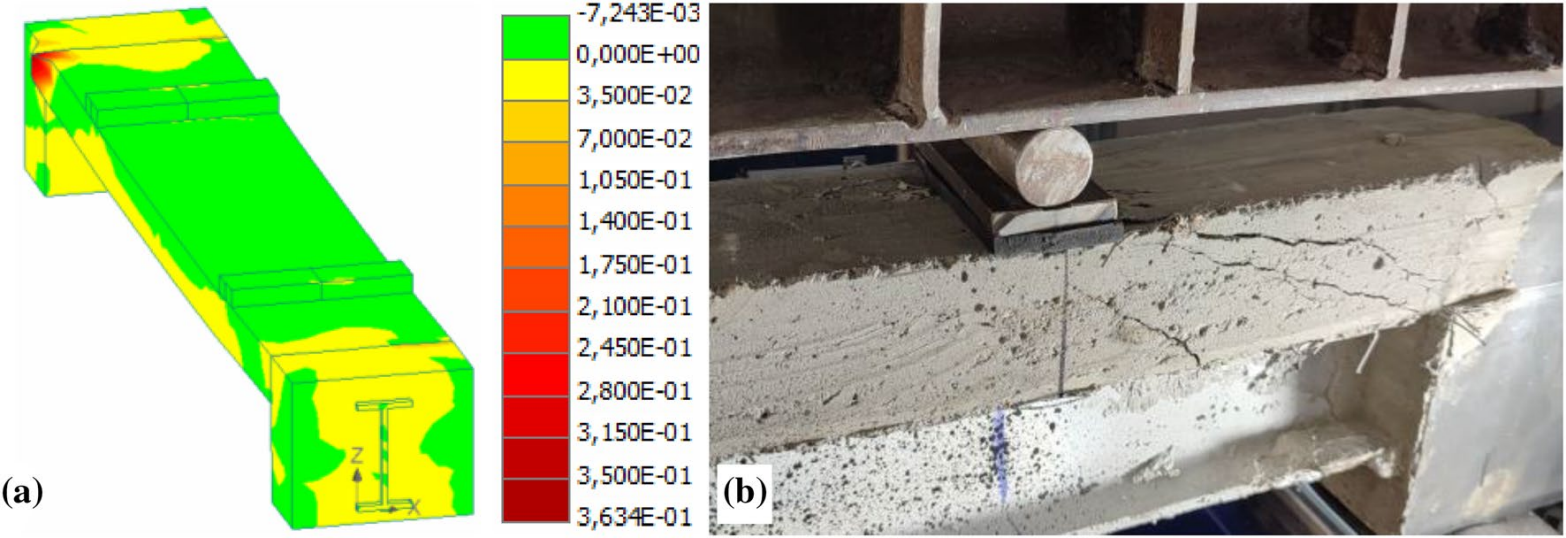
Failure mechanisms of the hybrid beams: (**a**) The predicted strain distribution in the FE model with the perfect bond (Fig. 6a); (**b**) Shear failure of the concrete in the second series beam.

The FRP failure simulations with Atena^7,29^ provided similar outcomes. Li et al.^34^ and Zhu et al.^12^ described a possible solution, presenting the progressive damage model for laminated composites. However, its application for the analysis of pultruded materials is still limited. On the contrary, modifying the support joints simplified the failure prediction problem. For instance, Fig. 9 shows the failure localization process predicted by Atena that corresponds to the deformation results shown in Fig. 6a. This example provides insight for further development of the hybrid beam systems, which modify the stress-ribbon concept for efficient utilization of advanced composite materials.

## Methods

**Figure 10:**
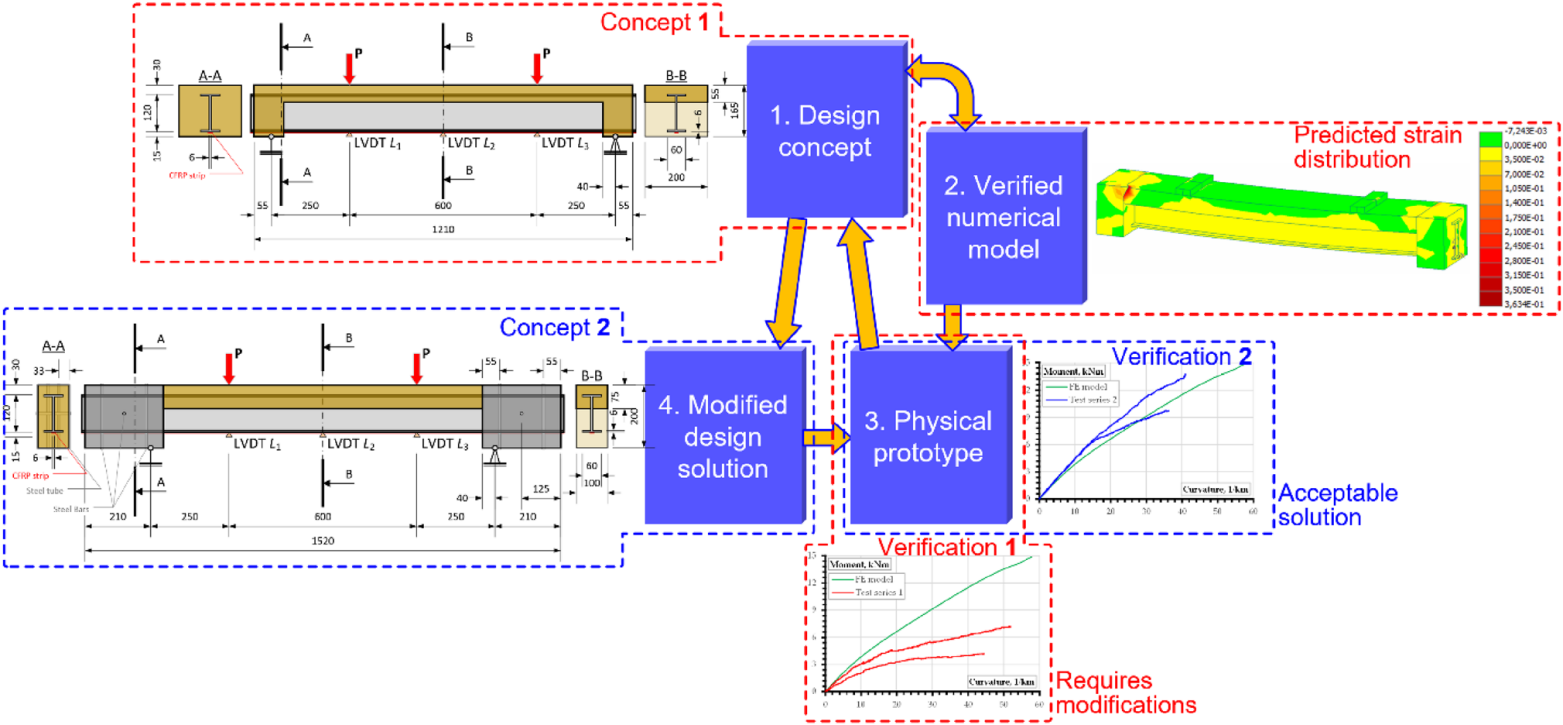
The adaptive design concept.

This study presents a novel design concept of the hybrid beam system comprising the synthetic fiber-reinforced concrete slab and pultruded FRP profile fixed on the supports. Adapting the stress-ribbon structural approach^15,30^ allows for solving the bond issue and applying the simplified numerical model, assuming the perfect bond between the composite components. The considered case exemplifies the design of the hybrid systems (Fig. 1) when the FE modeling outcome (Fig. 6a) defines the objective reference for the design procedure describing the hybrid system efficiency and modifies the structural target (Fig. 3). Figure 10 schematizes the proposed concept.

On the one hand, the proposed structural solution contradicts the traditional idea of local bond improvement, e.g., employing FRP profile perforation and mechanical anchorage systems (e.g., references^16,17^). On the contrary, this study demonstrates that the solution to the support problem (resulting from a low resistance of pultruded FRP profiles to transverse loads) improves the structural performance of the bridge prototype, doubling the structure’s flexural stiffness and load-bearing capacity regarding the weak concrete supports’ system.

On the other hand, the present study demonstrates the structural development concept when the FE modeling outcome defines the target reference of the design procedure. As Fig. 10 shows, the preliminary design concept (“1”) produces the numerical model (“2”), with the predicted parameters determining the efficient structural target. Further physical tests (“3”) check the concept “1” viability (i.e., “Verification 1”). If necessary, an engineer modifies the design solution (“4”). The iterative adaptation continues until the acceptable agreement between the physical and numerical outcomes is achieved (e.g., “Verification 2”).

## Conclusions

This study introduces a novel hybrid beam system’s design concept when the numerically predicted outcome modifies the design target. The numerical simulations and physical experiments prove the viability of the proposed idea. The following significant conclusions result from this study:

- Adapting the stress-ribbon structural approach allows for solving the bond-slip problem between the composite components, simplifying the numerical model of the hybrid system.
- The proposed modification of the anchor blocks ensures reliable fixing of the GFRP profile. In addition, it solves the FRP vulnerability problem to the loads acting in the transverse direction to the pultrusion pathway.
- The considered adaptive design concept, when an experimentally verified numerical model describes the structural design target, demonstrates the room for practical applications, doubling the structure’s flexural stiffness and load-bearing capacity regarding the weak concrete supports’ system. However, additional tests are necessary to ensure the outcomes’ reliability and optimize the support blocks’ geometry.
- The developed numerical (finite element) model determines the adequate reference for designing the hybrid structural systems and efficiently utilizing advanced composite materials.

## Data Availability

The analyzed datasets in this study are available from the corresponding author upon reasonable request.
